# *RAN/RANBP2* polymorphisms and neuroblastoma risk in Chinese children: a three-center case-control study

**DOI:** 10.18632/aging.101429

**Published:** 2018-04-28

**Authors:** Juxiang Wang, Zhenjian Zhuo, Min Chen, Jinhong Zhu, Jie Zhao, Jiao Zhang, Shanshan Chen, Jing He, Haixia Zhou

**Affiliations:** 1 Department of Hematology, The Second Affiliated Hospital and Yuying Children's Hospital of Wenzhou Medical University, Wenzhou 325027, Zhejiang, China; 2 School of Chinese Medicine, Faculty of Medicine, The Chinese University of Hong Kong, Hong Kong 999077, China; 3 Department of Clinical Laboratory, Molecular Epidemiology Laboratory, Harbin Medical University Cancer Hospital, Harbin 150040, Heilongjiang, China; 4 Department of Pediatric Surgery, the First Affiliated Hospital of Zhengzhou University, Zhengzhou 450052, Henan, China; 5 Department of Pediatric Surgery, Guangzhou Institute of Pediatrics, Guangzhou Women and Children’s Medical Center, Guangzhou Medical University, Guangzhou, Guangdong 510623, China; * Equal contribution

**Keywords:** neuroblastoma, RAN, RANBP2, polymorphism, susceptibility

## Abstract

The genetic etiology of sporadic neuroblastoma remains largely obscure. *RAN* and *RANBP2* genes encode Ras-related nuclear protein and Ran-binding protein 2, respectively. These two proteins form Ran-RanBP2 complex that regulate various cellular activities including nuclear transport. Aberrant functions of the two proteins are implicated in carcinogenesis. Given the unknown role of *RAN/RANBP2* single nucleotide polymorphisms (SNPs) in neuroblastoma risk, we performed a multi-center case-control study in Chinese children to assess the association of the *RAN/RANBP2* SNPs with neuroblastoma risk. We analyzed three potentially functional SNPs in *RAN* gene (rs56109543 C>T, rs7132224 A>G, rs14035 C>T) and one in *RANBP2* (rs2462788 C>T) in 429 cases and 884 controls. Odds ratios (ORs) and 95% confidence intervals (CIs) were used to access the association between these four polymorphisms and neuroblastoma risk. No single variant was found to statistically significantly associate with neuroblastoma risk. However, individuals with 3 protective genotypes were less likely to develop neuroblastoma, in comparison to non-carriers (adjusted OR=0.33; 95% CI=0.12-0.96; *P*=0.042), as well as those with 0-2 protective genotypes (adjusted OR=0.33; 95% CI=0.11-0.94; *P*=0.038). Stratified analysis revealed no significant association for any of the four polymorphisms. Further studies are warranted to validate the weak impact of *RAN/RANBP2* SNPs on neuroblastoma risk.

## Introduction

Neuroblastoma is a common extracranial solid tumor that derives from neural crest progenitor cells [1,2]. Neuroblastoma mostly takes place in children younger than 1 year, and the average diagnosis time is about 17 months of age [3]. Neuroblastoma is characterized by a wide range of variable prognosis, spanning from spontaneous regression without chemotherapy to life-threatening tumor progression despite intensive treatment [4–7]. Approximately 50% of neuroblastomas behave in highly malignant fashion, with distant metastasis at the time of diagnosis [8,9]. Their 5-year survival rates remain less than 40% despite intensive, multi-modal therapy [10].

The affects of environmental factors on the risk of neuroblastoma have been investigated but remains undefined [11,12]. Growing evidence has been directed to the genetic factors predisposing patients to neuroblastoma. Familial neuroblastoma is largely attributed to germline mutations in *PHOX2B* [13] or *ALK* [14,15] gene. In contrast, the etiology of sporadic neuroblastoma, the most common type of neuroblastoma, remains partially unveiled. Several genome-wide association studies (GWASs) and the subsequent replication studies identified a number of neuroblastoma susceptibility alleles, including *BARD1*, *LIN28B*, *HACE1*, *LMO1*, *MMP20* and *CASC15* genes [16–23]. Moreover, candidate gene approaches also detected the genetic associations of *NEFL* [24] and *CDKN1B* [25] gene polymorphisms with neuroblastoma susceptibility.

Ran (Ras-related nuclear protein) is a small Ras-related GTP-binding protein. Ran mainly locates in the nucleus and cycles between the GDP-bound inactive and the GTP-bound active state [26]. It facilitates the movement of molecules in and out of the nuclear-pore complexes [27]. Dysregulated protein level of Ran could cause aberrant nuclear-cytoplasmic transport of tumor suppressors and oncogenes, which might lead to the initiation of cancer [28]. Moreover, Ran also mediates several crucial functions, such as promoting spindle assembly, regulating cell cycle, and facilitating pre-mRNA generation [29]. RanBP2 (Ran-binding protein 2) is the largest protein of the nuclear pore complex (350 kDa). It contains rich FG-repeats, four Ran-binding domains and binds to Ran GTP with high affinity [30]. RanBP2 was initially described to be implicated in regulating nuclear transport due to its linkage with Ran [31]. It was further identified to regulate numerous cellular activities [32–34]. *RAN/RANBP2* genes are reported to be associated with cancer development. However, the association of polymorphisms in the *RAN/RANBP2* genes and neuroblastoma risk has yet to be elucidated. To address this issue, we conducted a three-center case-control study in a Chinese population.

## RESULTS

### Characteristics of study population

The detailed characteristics of subjects from Guangzhou and Zhengzhou were provided in the previous publications [35–37]. The detailed demographic characteristics in neuroblastoma patients and controls for Wenzhou, Guangdong and Henan subjects were presented in Supplementary Table 1. There were no significant differences between cases and controls from Wenzhou regarding age (20.25 ± 20.73 vs. 23.58 ± 15.36 months old, *P*=0.496) and gender (*P*=1.000).

### *RAN/RANBP2* polymorphisms and neuroblastoma risk

The genotype frequencies of *RAN/RANBP2* genes polymorphisms (Supplementary Table 2) and neuroblastoma susceptibility between all cases and controls were presented in Table 1 and Supplementary Table 3. All genotype frequencies in controls were in Hardy-Weinberg equilibrium (HWE) (rs56109543, *P*=0.587; rs7132224, *P*=0.289; rs14035, *P*=0.800; rs2462788, *P*=0.194). In single locus analysis, no statistically significant association were found regarding all the four SNPs and neuroblastoma risk. We further investigated the combined effect of protective genotypes of *RAN* in neuroblastoma risk. We observed that individuals with 3 protective genotypes were at significantly lower risk of developing neuroblastoma than those without protective genotypes [adjusted odds ratio (OR)=0.33; 95% confidence interval (CI)=0.12-0.96; *P*=0.042]. Moreover, subjects with 3 combined risk genotypes of *RAN* have a significant decreased risk of neuroblastoma (adjusted OR=0.33; 95% CI=0.11-0.94; *P*=0.038), compared with those with 0-2 protective genotypes.

**Table 1:** Association of *RAN* and *RANBP2* polymorphisms with neuroblastoma risk.

Genotype	Cases (N=429)	Controls (N=884)	*P* ^a^	Crude OR (95% CI)	*P*	Adjusted OR (95% CI) ^b^	*P* ^b^
*RAN* rs56109543 (HWE=0.587)							
CC	304 (70.86)	620 (70.14)		1.00		1.00	
CT	118 (27.51)	238 (26.92)		1.01 (0.78-1.31)	0.933	1.01 (0.78-1.31)	0.942
TT	7 (1.63)	26 (2.94)		0.55 (0.24-1.28)	0.165	0.55 (0.24-1.29)	0.168
Additive			0.363	0.93 (0.74-1.16)	0.504	0.93 (0.74-1.16)	0.502
Dominant	125 (29.14)	264 (29.86)	0.787	0.97 (0.75-1.24)	0.787	0.97 (0.75-1.24)	0.781
Recessive	422 (98.37)	858 (97.06)	0.155	0.55 (0.24-1.27)	0.161	0.55 (0.24-1.28)	0.164
*RAN* rs7132224 (HWE=0.289)							
AA	227 (52.91)	479 (54.19)		1.00		1.00	
AG	170 (39.63)	335 (37.90)		1.07 (0.84-1.37)	0.581	1.07 (0.84-1.36)	0.596
GG	32 (7.46)	70 (7.92)		0.97 (0.62-1.51)	0.875	0.96 (0.62-1.51)	0.870
Additive			0.823	1.02 (0.85-1.22)	0.828	1.02 (0.85-1.22)	0.842
Dominant	202 (47.09)	405 (45.81)	0.665	1.05 (0.84-1.33)	0.665	1.05 (0.83-1.32)	0.680
Recessive	397 (92.54)	814 (92.08)	0.771	0.94 (0.61-1.45)	0.771	0.94 (0.61-1.45)	0.770
*RAN* rs14035 (HWE=0.800)							
CC	285 (66.43)	590 (66.74)		1.00		1.00	
CT	135 (31.47)	263 (29.75)		1.06 (0.83-1.37)	0.635	1.06 (0.83-1.37)	0.641
TT	9 (2.10)	31 (3.51)		0.60 (0.28-1.28)	0.187	0.60 (0.28-1.29)	0.191
Additive			0.338	0.96 (0.78-1.19)	0.731	0.96 (0.78-1.19)	0.727
Dominant	144 (33.57)	294 (33.26)	0.912	1.01 (0.79-1.30)	0.911	1.01 (0.79-1.29)	0.918
Recessive	420 (97.90)	853 (96.49)	0.164	0.59 (0.28-1.25)	0.169	0.59 (0.28-1.26)	0.173
*RANBP2* rs2462788 (HWE=0.194)							
CC	402 (93.71)	810 (91.63)		1.00		1.00	
CT	27 (6.29)	74 (8.37)		0.74 (0.47-1.16)	0.187	0.74 (0.47-1.16)	0.188
TT	0 (0.00)	0 (0.00)		/	/	/	/
Additive			0.185	0.74 (0.47-1.16)	0.187	0.74 (0.47-1.16)	0.188
Dominant	27 (6.29)	74 (8.37)	0.185	0.74 (0.47-1.16)	0.187	0.74 (0.47-1.16)	0.188
Combined effect of protective genotypes for *RAN* ^c^							
0	394 (91.84)	814 (92.08)	0.073 ^d^	1.00		1.00	
1	26 (6.06)	38 (4.30)		1.41 (0.85-2.36)	0.186	1.41 (0.84-2.36)	0.189
2	5 (1.17)	7 (0.79)		1.48 (0.47-4.68)	0.509	1.48 (0.47-4.70)	0.507
3	4 (0.93)	25 (2.83)		**0.33 (0.11-0.96)**	**0.041**	**0.33 (0.12-0.96)**	**0.042**
0-2	425 (99.07)	859 (97.17)		1.00		1.00	
3	4 (0.93)	25 (2.83)	0.028	**0.32 (0.11-0.94)**	**0.037**	**0.33 (0.11-0.94)**	**0.038**

OR, odds ratio; CI, confidence interval; HWE, Hardy-Weinberg equilibrium.

^a^ *χ^2^* test for genotype distributions between neuroblastoma patients and controls.

^b^ Adjusted for age and gender.

^c^ Protective genotypes were rs56109543 TT, rs7132224 GG and rs14035 TT.

^d^ For additive model.

### Stratification analysis

Stratification analysis was further adopted to assess the effects of the *RAN* polymorphisms on neuroblastoma risk among different strata (Table 2). However, we failed to detect significant association for any of the four polymorphisms in single locus analysis. Moreover, the cumulative effects of protective genotypes were also insignificant.

**Table 2:** Stratification analysis for the association between *RAN* gene genotypes and neuroblastoma susceptibility.

Variables	rs56109543 (case/control)		AOR (95% CI) ^a^	*P* ^a^	rs14035 (case/control)		AOR (95% CI) ^a^	*P* ^a^	Protective genotypes (case/control)		AOR (95% CI) ^a^	*P* ^a^
	CC/CT	TT			CC/CT	TT			0-2	3		
Age, month												
≤18	145/327	1/13	0.17 (0.02-1.32)	0.091	144/326	2/14	0.33 (0.07-1.45)	0.140	145/328	1/12	0.19 (0.02-1.45)	0.109
>18	277/531	6/13	0.89 (0.33-2.35)	0.806	276/527	7/17	0.79 (0.32-1.92)	0.597	280/531	3/13	0.44 (0.12-1.55)	0.200
Gender												
Female	181/365	4/11	0.70 (0.22-2.24)	0.550	183/366	2/10	0.38 (0.08-1.77)	0.219	183/366	2/10	0.38 (0.08-1.77)	0.219
Male	241/493	3/15	0.42 (0.12-1.46)	0.170	237/487	7/21	0.68 (0.29-1.63)	0.392	242/493	2/15	0.28 (0.06-1.22)	0.090
Sites of origin												
Adrenal gland	163/858	1/26	0.22 (0.03-1.61)	0.134	160/853	4/31	0.71 (0.25-2.03)	0.518	164/859	0/25	/	/
Retroperitoneal	94/858	2/26	0.68 (0.16-2.92)	0.604	93/853	3/31	0.85 (0.25-2.83)	0.785	94/859	2/25	0.71 (0.16-3.03)	0.638
Mediastinum	119/858	4/26	1.08 (0.37-3.16)	0.887	121/853	2/31	0.46 (0.11-1.96)	0.295	121/859	2/25	0.56 (0.13-2.39)	0.432
Others	38/858	0/26	/	/	38/853	0/31	/	/	38/859	0/25	/	/
Clinical stage												
I+II+4s	175/858	4/26	0.73 (0.25-2.13)	0.567	176/853	3/31	0.47 (0.14-1.56)	0.217	177/859	2/25	0.38 (0.09-1.62)	0.190
III+IV	224/858	3/26	0.47 (0.14-1.59)	0.226	221/853	6/31	0.76 (0.31-1.86)	0.550	225/859	2/25	0.33 (0.08-1.39)	0.129

AOR, adjusted odds ratio; CI, confidence interval.

^a^ Adjusted for age and gender, omitting the corresponding stratification factor.

## DISCUSSION

In the current study, we performed the first investigation into the impact of SNPs in *RAN/RANBP2* genes on the risk of neuroblastoma in Chinese Han children. Our data revealed that the single *RAN* or *RANBP2* gene polymorphism might not be strong enough to confer the neuroblastoma susceptibility in Chinese children. However, three protective *RAN* genotypes were observed to cumulatively reduce the risk of neuroblastoma.

Overexpression of Ran has been observed in several human malignancies, including lung, prostate, breast, colon cancer, and neuroblastoma [38,39]. Conditional knockdown of *RAN* gene reduced the viability of activated K-Ras-transformed cells, through inducing S-phase arrest [40]. Barrès et al. found that Ran protein is highly expressed in invasive serous epithelial ovarian cancers and overexpression of Ran is associated with poor patient outcome [41]. They also detected that silencing Ran could impair tumor growth *in vitro* and *in vivo* [42]. Xia et al. showed that RNA interference-mediated knockdown of *RAN* induces aberrant mitotic formation and apoptosis in cancer cells [38]. Silencing *RAN* causes abnormal nucleocytoplasmic transportation of transcription factors in tumor cells [43]. RanBP2 protein also plays critical roles in cellular processes. Knockdown of *RANBP2* results in an aberrant metaphase, mitotic arrest in G_2_/M phase and mitotic cell death [44]. A study by Dawlaty et al. demonstrated that RanBP2 acts as a novel tumor suppressor in lung cancer through regulating TopoII by sumoylation [45]. In addition, RanBP2 hypomorphic mice are more susceptible to spontaneous and carcinogen-induced lung tumors. Consistently, two independent studies also demonstrated that RanBP2 level was downregulated in human lung cancers [46,47].

Herein, for the first time we investigated whether *RAN/RANBP2* SNPs could contribute to the risk of neuroblastoma in Chinese children. However, our findings found no significant relationship between all the analyzed *RAN/RANBP2* polymorphisms and neuroblastoma risk. Such null relationship might be attributed to the relatively small sample size, although we tried to expand the sample by recruiting subjects from three centers. To be highlighted, a study conducted by Luo et al. explored the association between sumoylation-related genes polymorphisms and risk of gastric cancer [48]. They are the first group investigating the role of *RANBP2* gene polymorphism in cancer risk. Their study included 1021 gastric cancer cases and 1304 controls from Chinese population. However, they failed to obtain a significant association between *RANBP2* gene intron variant rs12614691 and gastric cancer risk. In the combined analysis of our study, subjects carrying 3 protective genotypes tend to have decreased neuroblastoma risk in comparison to those without risk genotypes or those with 0-2 protective genotypes. This phenomenon was quite biologically plausible as each single variant in each gene might not be strong enough to influence the risk of cancer.

The current study was the first investigation on the association of *RAN/RANBP2* genes SNPs with neuroblastoma risk. Another merit of this study was that this is a three-center case-control study. Several limitations exist in the current study. First, because of the low incidence rate of neuroblastoma, the recruitment of eligible patients was a great challenge for us. Even though we enrolled participants from three hospitals, the sample size is still moderate. This limited sample size inevitably impaired the strength of the statistical power. Second, this study only incorporated four SNPs in the *RAN/RANBP2* genes. Future studies should investigate more potentially functional polymorphisms in *RAN/RANBP2* genes. Third, as all the participants included were of Chinese origin, conclusions should be taken with caution when extrapolated to other populations. Fourth, functional analysis is warranted to justify the described associations, which would illustrate the underlying mechanisms of how theses SNPs modify neuroblastoma susceptibility. Additionally, we only assessed the possible association of the SNPs with neuroblastoma risk. Other environmental factors, such as dietary habit, childhood exposure, and health situation, would help to provide further insight into the influence of *RAN/RANBP2* polymorphisms on neuroblastoma risk.

In all, here we demonstrate that common variants at the *RAN/RANBP2* genes are associated with the risk of neuroblastoma in the Chinese children in a low-impact manner. Future larger-sample, functional studies are warranted to address the mechanism by which *RAN/RANBP2* SNPs impacts tumorigenesis of neuroblastoma.

## MATERIALS AND METHODS

### Study populations

This case-control study was conducted in three centers: Guangzhou Women and Children’s Medical Center, The First Affiliated Hospital of Zhengzhou University and The Second Affiliated Hospital and Yuying Children's Hospital of Wenzhou Medical University. The study was approved by the Institutional Review Board of the above three hospitals. In total, 429 neuroblastoma cases and 884 controls from three centers were included in this study. To be specific, 275 cases and 531 controls were enrolled from Guangzhou [35–37], 118 cases and 281 controls were recruited from Zhengzhou [49,50], and 36 cases and 72 controls were enrolled from Wenzhou (Supplementary Table 1). The recruitment period lasts from December 2007 to June 2017. All the participants’ parents provided signed informed consent before the study. Selection criteria of the included participants were accessible in our previous publication [51].

### SNP selection and genotyping

We chose potentially functional polymorphisms in the *RAN/RANBP2* genes from dbSNP database (http://www.ncbi.nlm.nih.gov/). An online tool, SNPinfo (http://snpinfo.niehs.nih.gov/) was used to predict the functions of SNPs. In brief, we searched the potentially functional candidate SNPs located in the 5’- flanking region, 5’ untranslated region, 3’ untranslated region, and exon of *RAN/RANBP2* genes. Three potentially functional SNPs in *RAN* gene (rs56109543 C>T, rs7132224 A>G, rs14035 C>T) and one SNP in *RANBP2* (rs2462788 C>T) were chosen for analysis that captured nine additional SNPs with LD>0.8 (Supplementary Table 2). Three SNPs (rs56109543, rs7132224, rs2462788) are located in transcription factor binding sites (TFBS) and one SNP rs14035 might affect the microRNA binding site activity. As shown in Supplementary Figure 1, there was no significant LD (R^2^<0.8) between each *RAN* SNP pair (R^2^=0.488 between rs56109543 and rs7132224, R^2^=0.582 between rs14035 and rs7132224), except for the rs56109543 and rs14035 (R^2^=0.838).

The peripheral blood was used to extract genomic DNA. We genotyped the gene polymorphisms using Taqman real-time PCR [52–54]. On each 384-well plate, eight negative controls with water were used as quality control samples. The randomized and blinded process method was adopted to genotype all case and control samples. 10% random selection samples were re-genotyped and the genotype concordance rate was 100%.

### Statistical analysis

Departures from HWE for the selected SNPs in controls was evaluated using goodness-of-fit χ^2^ test. Allele frequencies and demographic variables between the two groups were assessed by chi-square test. The ORs, 95% CIs, and the corresponding *P* value for each SNP were calculated with adjustment for age and gender. Risk associations between genotypes and neuroblastoma were determined from logistic regression analysis. All calculations were performed using SAS software version 9.4 (SAS Institute, Cary, NC). All statistical tests were two-sided, and significant threshold was set using *P*< 0.05.

## Supplementary Material

Supplementary File

## References

[1] Matthay KK, Maris JM, Schleiermacher G, Nakagawara A, Mackall CL, Diller L, Weiss WA. Neuroblastoma. Nat Rev Dis Primers. 2016; 2:16078. 10.1038/nrdp.2016.78 27830764

[2] Capasso M, Diskin SJ. Genetics and genomics of neuroblastoma. Cancer Treat Res. 2010; 155:65–84. 10.1007/978-1-4419-6033-7_4 20517688

[3] Cheung NK, Dyer MA. Neuroblastoma: developmental biology, cancer genomics and immunotherapy. Nat Rev Cancer. 2013; 13:397–411. 10.1038/nrc3526 23702928 PMC4386662

[4] Maris JM. Recent advances in neuroblastoma. N Engl J Med. 2010; 362:2202–11. 10.1056/NEJMra0804577 20558371 PMC3306838

[5] Schwab M, Westermann F, Hero B, Berthold F. Neuroblastoma: biology and molecular and chromosomal pathology. Lancet Oncol. 2003; 4:472–80. 10.1016/S1470-2045(03)01166-5 12901961

[6] Irwin MS, Park JR. Neuroblastoma: paradigm for precision medicine. Pediatr Clin North Am. 2015; 62:225–56. 10.1016/j.pcl.2014.09.015 25435121

[7] DuBois SG, Kalika Y, Lukens JN, Brodeur GM, Seeger RC, Atkinson JB, Haase GM, Black CT, Perez C, Shimada H, Gerbing R, Stram DO, Matthay KK. Metastatic sites in stage IV and IVS neuroblastoma correlate with age, tumor biology, and survival. J Pediatr Hematol Oncol. 1999; 21:181–89. 10.1097/00043426-199905000-00005 10363850

[8] Esposito MR, Aveic S, Seydel A, Tonini GP. Neuroblastoma treatment in the post-genomic era. J Biomed Sci. 2017; 24:14. 10.1186/s12929-017-0319-y 28178969 PMC5299732

[9] Park JR, Bagatell R, Cohn SL, Pearson AD, Villablanca JG, Berthold F, Burchill S, Boubaker A, McHugh K, Nuchtern JG, London WB, Seibel NL, Lindwasser OW, et al. Revisions to the International Neuroblastoma Response Criteria: A Consensus Statement From the National Cancer Institute Clinical Trials Planning Meeting. J Clin Oncol. 2017; 35:2580–87. 10.1200/JCO.2016.72.0177 28471719 PMC5676955

[10] Maris JM, Hogarty MD, Bagatell R, Cohn SL. Neuroblastoma. Lancet. 2007; 369:2106–20. 10.1016/S0140-6736(07)60983-0 17586306

[11] Cook MN, Olshan AF, Guess HA, Savitz DA, Poole C, Blatt J, Bondy ML, Pollock BH. Maternal medication use and neuroblastoma in offspring. Am J Epidemiol. 2004; 159:721–31. 10.1093/aje/kwh108 15051581

[12] Menegaux F, Olshan AF, Neglia JP, Pollock BH, Bondy ML. Day care, childhood infections, and risk of neuroblastoma. Am J Epidemiol. 2004; 159:843–51. 10.1093/aje/kwh111 15105177 PMC2080646

[13] Mosse YP, Laudenslager M, Khazi D, Carlisle AJ, Winter CL, Rappaport E, Maris JM. Germline PHOX2B mutation in hereditary neuroblastoma. Am J Hum Genet. 2004; 75:727–30. 10.1086/424530 15338462 PMC1182065

[14] Chen Y, Takita J, Choi YL, Kato M, Ohira M, Sanada M, Wang L, Soda M, Kikuchi A, Igarashi T, Nakagawara A, Hayashi Y, Mano H, Ogawa S. Oncogenic mutations of ALK kinase in neuroblastoma. Nature. 2008; 455:971–74. 10.1038/nature07399 18923524

[15] Mossé YP, Laudenslager M, Longo L, Cole KA, Wood A, Attiyeh EF, Laquaglia MJ, Sennett R, Lynch JE, Perri P, Laureys G, Speleman F, Kim C, et al. Identification of ALK as a major familial neuroblastoma predisposition gene. Nature. 2008; 455:930–35. 10.1038/nature07261 18724359 PMC2672043

[16] Capasso M, Devoto M, Hou C, Asgharzadeh S, Glessner JT, Attiyeh EF, Mosse YP, Kim C, Diskin SJ, Cole KA, Bosse K, Diamond M, Laudenslager M, et al. Common variations in BARD1 influence susceptibility to high-risk neuroblastoma. Nat Genet. 2009; 41:718–23. 10.1038/ng.374 19412175 PMC2753610

[17] He J, Wang F, Zhu J, Zhang Z, Zou Y, Zhang R, Yang T, Xia H. The *TP53* gene rs1042522 C>G polymorphism and neuroblastoma risk in Chinese children. Aging (Albany NY). 2017; 9:852–59. 10.18632/aging.101196 28275206 PMC5391235

[18] He J, Zhong W, Zeng J, Zhu J, Zhang R, Wang F, Yang T, Zou Y, Xia H. LMO1 gene polymorphisms contribute to decreased neuroblastoma susceptibility in a Southern Chinese population. Oncotarget. 2016; 7:22770–78. 10.18632/oncotarget.8178 27009839 PMC5008399

[19] Maris JM, Mosse YP, Bradfield JP, Hou C, Monni S, Scott RH, Asgharzadeh S, Attiyeh EF, Diskin SJ, Laudenslager M, Winter C, Cole KA, Glessner JT, et al. Chromosome 6p22 locus associated with clinically aggressive neuroblastoma. N Engl J Med. 2008; 358:2585–93. 10.1056/NEJMoa0708698 18463370 PMC2742373

[20] Diskin SJ, Capasso M, Diamond M, Oldridge DA, Conkrite K, Bosse KR, Russell MR, Iolascon A, Hakonarson H, Devoto M, Maris JM. Rare variants in TP53 and susceptibility to neuroblastoma. J Natl Cancer Inst. 2014; 106:dju047. 10.1093/jnci/dju047 24634504 PMC3982892

[21] Diskin SJ, Capasso M, Schnepp RW, Cole KA, Attiyeh EF, Hou C, Diamond M, Carpenter EL, Winter C, Lee H, Jagannathan J, Latorre V, Iolascon A, et al. Common variation at 6q16 within HACE1 and LIN28B influences susceptibility to neuroblastoma. Nat Genet. 2012; 44:1126–30. 10.1038/ng.2387 22941191 PMC3459292

[22] He J, Zhang R, Zou Y, Zhu J, Yang T, Wang F, Xia H. Evaluation of GWAS-identified SNPs at 6p22 with neuroblastoma susceptibility in a Chinese population. Tumour Biol. 2016; 37:1635–39. 10.1007/s13277-015-3936-7 26307394

[23] Chang X, Zhao Y, Hou C, Glessner J, McDaniel L, Diamond MA, Thomas K, Li J, Wei Z, Liu Y, Guo Y, Mentch FD, Qiu H, et al. Common variants in MMP20 at 11q22.2 predispose to 11q deletion and neuroblastoma risk. Nat Commun. 2017; 8:569. 10.1038/s41467-017-00408-8 28924153 PMC5603517

[24] Capasso M, Diskin S, Cimmino F, Acierno G, Totaro F, Petrosino G, Pezone L, Diamond M, McDaniel L, Hakonarson H, Iolascon A, Devoto M, Maris JM. Common genetic variants in NEFL influence gene expression and neuroblastoma risk. Cancer Res. 2014; 74:6913–24. 10.1158/0008-5472.CAN-14-0431 25312269 PMC4253722

[25] Capasso M, McDaniel LD, Cimmino F, Cirino A, Formicola D, Russell MR, Raman P, Cole KA, Diskin SJ. The functional variant rs34330 of CDKN1B is associated with risk of neuroblastoma. J Cell Mol Med. 2017; 21:3224–30. 10.1111/jcmm.13226 28667701 PMC5706517

[26] Bischoff FR, Krebber H, Smirnova E, Dong W, Ponstingl H. Co-activation of RanGTPase and inhibition of GTP dissociation by Ran-GTP binding protein RanBP1. EMBO J. 1995; 14:705–15. 7882974 10.1002/j.1460-2075.1995.tb07049.x PMC398135

[27] Görlich D, Kutay U. Transport between the cell nucleus and the cytoplasm. Annu Rev Cell Dev Biol. 1999; 15:607–60. 10.1146/annurev.cellbio.15.1.607 10611974

[28] Kau TR, Way JC, Silver PA. Nuclear transport and cancer: from mechanism to intervention. Nat Rev Cancer. 2004; 4:106–17. 10.1038/nrc1274 14732865

[29] Clarke PR, Zhang C. Spatial and temporal coordination of mitosis by Ran GTPase. Nat Rev Mol Cell Biol. 2008; 9:464–77. 10.1038/nrm2410 18478030

[30] Pichler A, Gast A, Seeler JS, Dejean A, Melchior F. The nucleoporin RanBP2 has SUMO1 E3 ligase activity. Cell. 2002; 108:109–20. 10.1016/S0092-8674(01)00633-X 11792325

[31] Melchior F, Guan T, Yokoyama N, Nishimoto T, Gerace L. GTP hydrolysis by Ran occurs at the nuclear pore complex in an early step of protein import. J Cell Biol. 1995; 131:571–81. 10.1083/jcb.131.3.571 7593180 PMC2120626

[32] Ferreira PA, Nakayama TA, Pak WL, Travis GH. Cyclophilin-related protein RanBP2 acts as chaperone for red/green opsin. Nature. 1996; 383:637–40. 10.1038/383637a0 8857542

[33] Aslanukov A, Bhowmick R, Guruju M, Oswald J, Raz D, Bush RA, Sieving PA, Lu X, Bock CB, Ferreira PA. RanBP2 modulates Cox11 and hexokinase I activities and haploinsufficiency of RanBP2 causes deficits in glucose metabolism. PLoS Genet. 2006; 2:e177. 10.1371/journal.pgen.0020177 17069463 PMC1626108

[34] Gloerich M, Vliem MJ, Prummel E, Meijer LA, Rensen MG, Rehmann H, Bos JL. The nucleoporin RanBP2 tethers the cAMP effector Epac1 and inhibits its catalytic activity. J Cell Biol. 2011; 193:1009–20. 10.1083/jcb.201011126 21670213 PMC3115801

[35] He J, Zou Y, Liu X, Zhu J, Zhang J, Zhang R, Yang T, Xia H. Association of Common Genetic Variants in Pre-microRNAs and Neuroblastoma Susceptibility: A Two-Center Study in Chinese Children. Mol Ther Nucleic Acids. 2018; 11:1–8. 10.1016/j.omtn.2018.01.003 PMC5849804 29858046

[36] Zhang Z, Chang Y, Jia W, Zhang J, Zhang R, Zhu J, Yang T, Xia H, Zou Y, He J. *LINC00673* rs11655237 C>T confers neuroblastoma susceptibility in Chinese population. Biosci Rep. 2018; 38:BSR20171667. 10.1042/BSR20171667 29339420 PMC5803493

[37] Zhuo ZJ, Liu W, Zhang J, Zhu J, Zhang R, Tang J, Yang T, Zou Y, He J, Xia H. Functional Polymorphisms at ERCC1/XPF Genes Confer Neuroblastoma Risk in Chinese Children. EBioMedicine. 2018; 30:113–19. 10.1016/j.ebiom.2018.03.003 29544698 PMC5952228

[38] Xia F, Lee CW, Altieri DC. Tumor cell dependence on Ran-GTP-directed mitosis. Cancer Res. 2008; 68:1826–33. 10.1158/0008-5472.CAN-07-5279 18339863

[39] Schnepp RW, Khurana P, Attiyeh EF, Raman P, Chodosh SE, Oldridge DA, Gagliardi ME, Conkrite KL, Asgharzadeh S, Seeger RC, Madison BB, Rustgi AK, Maris JM, Diskin SJ. A LIN28B-RAN-AURKA Signaling Network Promotes Neuroblastoma Tumorigenesis. Cancer Cell. 2015; 28:599–609. 10.1016/j.ccell.2015.09.012 26481147 PMC4643330

[40] Morgan-Lappe SE, Tucker LA, Huang X, Zhang Q, Sarthy AV, Zakula D, Vernetti L, Schurdak M, Wang J, Fesik SW. Identification of Ras-related nuclear protein, targeting protein for xenopus kinesin-like protein 2, and stearoyl-CoA desaturase 1 as promising cancer targets from an RNAi-based screen. Cancer Res. 2007; 67:4390–98. 10.1158/0008-5472.CAN-06-4132 17483353

[41] Ouellet V, Guyot MC, Le Page C, Filali-Mouhim A, Lussier C, Tonin PN, Provencher DM, Mes-Masson AM. Tissue array analysis of expression microarray candidates identifies markers associated with tumor grade and outcome in serous epithelial ovarian cancer. Int J Cancer. 2006; 119:599–607. 10.1002/ijc.21902 16572426

[42] Barrès V, Ouellet V, Lafontaine J, Tonin PN, Provencher DM, Mes-Masson AM. An essential role for Ran GTPase in epithelial ovarian cancer cell survival. Mol Cancer. 2010; 9:272. 10.1186/1476-4598-9-272 20942967 PMC2964620

[43] Yuen HF, Chan KK, Grills C, Murray JT, Platt-Higgins A, Eldin OS, O’Byrne K, Janne P, Fennell DA, Johnston PG, Rudland PS, El-Tanani M. Ran is a potential therapeutic target for cancer cells with molecular changes associated with activation of the PI3K/Akt/mTORC1 and Ras/MEK/ERK pathways. Clin Cancer Res. 2012; 18:380–91. 10.1158/1078-0432.CCR-11-2035 22090358 PMC3272446

[44] Hashizume C, Kobayashi A, Wong RW. Down-modulation of nucleoporin RanBP2/Nup358 impaired chromosomal alignment and induced mitotic catastrophe. Cell Death Dis. 2013; 4:e854. 10.1038/cddis.2013.370 24113188 PMC3824679

[45] Dawlaty MM, Malureanu L, Jeganathan KB, Kao E, Sustmann C, Tahk S, Shuai K, Grosschedl R, van Deursen JM. Resolution of sister centromeres requires RanBP2-mediated SUMOylation of topoisomerase IIalpha. Cell. 2008; 133:103–15. 10.1016/j.cell.2008.01.045 18394993 PMC2693193

[46] Beer DG, Kardia SL, Huang CC, Giordano TJ, Levin AM, Misek DE, Lin L, Chen G, Gharib TG, Thomas DG, Lizyness ML, Kuick R, Hayasaka S, et al. Gene-expression profiles predict survival of patients with lung adenocarcinoma. Nat Med. 2002; 8:816–24. 10.1038/nm733 12118244

[47] Garber ME, Troyanskaya OG, Schluens K, Petersen S, Thaesler Z, Pacyna-Gengelbach M, van de Rijn M, Rosen GD, Perou CM, Whyte RI, Altman RB, Brown PO, Botstein D, Petersen I. Diversity of gene expression in adenocarcinoma of the lung. Proc Natl Acad Sci USA. 2001; 98:13784–89. 10.1073/pnas.241500798 11707590 PMC61119

[48] Luo Y, You S, Wang J, Fan S, Shi J, Peng A, Yu T. Association between Sumoylation-Related Gene rs77447679 Polymorphism and Risk of Gastric Cancer (GC) in a Chinese Population. J Cancer. 2017; 8:3226–31. 10.7150/jca.20587 29158794 PMC5665038

[49] Zhang J, Lin H, Wang J, He J, Zhang D, Qin P, Yang L, Yan L. *LMO1* polymorphisms reduce neuroblastoma risk in Chinese children: a two-center case-control study. Oncotarget. 2017; 8:65620–26. 10.18632/oncotarget.20018 29029458 PMC5630358

[50] Zhang J, Zhuo ZJ, Wang J, He J, Yang L, Zhang D, Qin P, Yan L. *CASC15* gene polymorphisms reduce neuroblastoma risk in Chinese children. Oncotarget. 2017; 8:91343–49. 10.18632/oncotarget.20514 29207648 PMC5710928

[51] He J, Wang F, Zhu J, Zhang R, Yang T, Zou Y, Xia H. Association of potentially functional variants in the XPG gene with neuroblastoma risk in a Chinese population. J Cell Mol Med. 2016; 20:1481–90. 10.1111/jcmm.12836 27019310 PMC4956948

[52] Gong J, Tian J, Lou J, Wang X, Ke J, Li J, Yang Y, Gong Y, Zhu Y, Zou D, Peng X, Yang N, Mei S, et al. A polymorphic MYC response element in KBTBD11 influences colorectal cancer risk, especially in interaction with a MYC regulated SNP rs6983267. Ann Oncol. 2018; 29:632–39. 10.1093/annonc/mdx789 29267898

[53] Lou J, Gong J, Ke J, Tian J, Zhang Y, Li J, Yang Y, Zhu Y, Gong Y, Li L, Chang J, Zhong R, Miao X. A functional polymorphism located at transcription factor binding sites, rs6695837 near LAMC1 gene, confers risk of colorectal cancer in Chinese populations. Carcinogenesis. 2017; 38:177–83. 28039327 10.1093/carcin/bgw204

[54] Zou D, Lou J, Ke J, Mei S, Li J, Gong Y, Yang Y, Zhu Y, Tian J, Chang J, Zhong R, Gong J, Miao X. Integrative expression quantitative trait locus-based analysis of colorectal cancer identified a functional polymorphism regulating SLC22A5 expression. Eur J Cancer. 2018; 93:1–9. 10.1016/j.ejca.2018.01.065 29428571

