# Supplementary material for: RAN/RANBP2 polymorphisms and neuroblastoma risk in Chinese children: a three-center case-control study
Source: Aging (Albany NY). 2018 Apr 28;10(4):808–18. doi: 10.18632/aging.101429 (PMC5940104; doi:10.18632/aging.101429)
Supplement: Supplementary File [file aging-10-101429-s001.pdf]

SUPPLEMENTARY MATERIAL

Supplementary Table 1. **Frequency distribution of selected characteristics in neuroblastoma patients and controls.**

Variables	Wenzhou					Guangdong province					Henan province				
	Cases (N=36)		Controls (N=72)		*P* [a]	Cases (N=275)		Controls (N=531)		*P* [a]	Cases (N=118)		Controls (N=281)		*P* [a]
	No.	%	No.	No.		No.	%	No.	%		No.	%	No.	%	
Age range, month	0.05-72		8-72		0.496	0-132		0.07-156		0.079	0-131.1		0.1-144.0		0.189
Mean ± SD	20.25±20.73		23.58±15.36			31.50±25.43		29.73±24.86			46.24±29.98		44.97±33.23		
≤18	20	55.56	35	48.61		103	37.45	233	43.88		23	19.49	72	25.62	
>18	16	44.44	37	51.39		172	62.55	298	56.12		95	80.51	209	74.38	
Gender					1.000					0.510					0.196
Female	17	47.22	34	47.22		114	41.45	233	43.88		54	45.76	109	38.79	
Male	19	52.78	38	52.78		161	58.55	298	56.12		64	54.24	172	61.21	
Clinical stages															
I	15	41.67	/	/		54	19.64				15	12.71			
II	2	5.56	/	/		62	22.55				31	26.27			
III	9	25.00	/	/		49	17.82				19	16.10			
IV	7	19.44	/	/		94	34.18				49	41.53			
4s	3	8.33	/	/		8	2.91				3	2.54			
NA						8	2.91				1	0.85			
Sites of origin															
Adrenal gland	11	30.56	/	/		64	23.27				89	75.42			
Retroperitoneal region	9	25.00	/	/		87	31.64				/	/			
Mediastinum	14	38.89	/	/		90	32.73				19	16.10			
Other region	2	5.56	/	/		26	9.45				10	8.47			
NA						8	2.91				/	/			

SD, standard deviation; NA, not available.

[a] Two-sided χ^2 test for distributions between neuroblastoma cases and cancer-free controls.

Supplementary Table 2. Polymorphisms captured by the four selected functional polymorphisms in *RAN/RANBP2* genes as predicted by SNPinfo (https://snpinfo.niehs.nih.gov/snpinfo/snpfunc.html).

rs	Chr.	Allele	LDsnp	Pop/LD	TFBS	miRNA(miRanda)	Nearby Gene	Allele	Asian	CHB
rs10773832	12	C/T	rs14035	CHB/0.957	--	--	*RAN*||*GPR133*	T	0.747	0.833
rs10773833	12	C/G	rs14035	CHB/0.957	--	--	*RAN*||*GPR133*	C	0.772	0.833
rs10848236	12	A/G	rs14035	CHB/0.87	Y	--	*STX2*||*RAN*	G	0.788	0.857
rs11061209	12	A/G	rs14035	CHB/0.957	--	--	*RAN*||*GPR133*	G	0.772	0.833
rs11061222	12	C/T	rs14035	CHB/0.957	--	--	*RAN*||*GPR133*	T	0.783	0.833
**rs14035**	**12**	**C/T**	**rs14035**	**1**	**--**	**Y**	***RAN***	**C**	**0.779**	**0.839**
rs3809142	12	C/T	rs14035	CHB/0.828	Y	--	*STX2*||*RAN*	C	--	0.863
rs7958223	12	A/C	rs14035	CHB/1	--	--	*RAN*	C	0.781	0.843
**rs2462788**	**2**	**T/C**	**rs2462788**	**1**	**Y**	**--**	***LOC644911*||*RANBP2***	**T**	**--**	**0.081**[a]
**rs56109543**	**12**	**C/T**	**rs56109543**	**1**	**Y**	**--**	***STX2*||*RAN***	**T**	**--**	**0.129** [a]
rs10848218	12	C/T	rs7132224	CHB/0.819	--	--	*STX2*||*RAN*	C	0.344	0.250
**rs7132224**	**12**	**A/G**	**rs7132224**	**1**	**Y**	**--**	***STX2*||*RAN***	**A**	**0.663**	**0.738**
rs7307055	12	G/T	rs7132224	CHB/0.936	--	--	*STX2*||*RAN*	G	0.683	0.759

SNP, single nucleotide polymorphism; LD, linkage disequilibrium; TFBS, transcription factor binding sites; CHB, Han Chinese in Beijing, China.

[a] Southern Han Chinese, using data from 1000 Genomes (https://www.ncbi.nlm.nih.gov/variation/tools/1000genomes/).

Supplementary Table 3. Association of *RAN* and *RANBP2* polymorphisms with neuroblastoma risk (Divided subjects)

Genotype	Guangdong province				Henan province				Wenzhou			
	Cases (N=275)	Controls (N=531)	AOR (95% CI) [a]	*P* [a]	Cases (N=118)	Controls (N=281)	AOR (95% CI) [a]	*P* [a]	Cases (N=36)	Controls (N=72)	AOR (95% CI) [a]	*P* [a]
RAN rs56109543 C>T												
CC	198 (72.00)	372 (70.06)	1.00		81 (68.64)	190 (67.62)	1.00		25 (69.44)	58 (80.56)	1.00	
CT	71 (25.82)	141 (26.55)	0.95 (0.68-1.32)	0.745	36 (30.51)	84 (29.89)	0.99 (0.62-1.58)	0.955	11 (30.56)	13 (18.06)	2.32 (0.87-6.3118)	0.094
TT	6 (2.18)	18 (3.39)	0.64 (0.25-1.64)	0.353	1 (0.85)	7 (2.49)	0.34 (0.04-2.85)	0.322	0 (0.00)	1 (1.39)	/	/
Additive			0.90 (0.68-1.19)	0.438			0.89 (0.58-1.37)	0.602			1.96 (0.77-4.97)	0.156
Dominant	77 (28.00)	159 (29.94)	0.91 (0.66-1.26)	0.576	37 (31.36)	91 (32.38)	0.94 (0.59-1.50)	0.790	11 (30.56)	14 (19.44)	2.23 (0.84-5.95)	0.108
Recessive	269 (97.82)	513 (96.61)	0.65 (0.26-1.66)	0.367	117 (99.15)	274 (97.51)	0.35 (0.04-2.85)	0.323	36 (100.00)	71 (98.61)	/	/
RAN rs7132224 A>G												
AA	148 (53.82)	291 (54.80)	1.00		59 (50.00)	142 (50.53)	1.00		20 (55.56)	46 (63.89)	1.00	
AG	109 (39.64)	199 (37.48)	1.08 (0.79-1.47)	0.627	48 (40.68)	115 (40.93)	0.99 (0.63-1.56)	0.975	13 (36.11)	21 (29.17)	1.58 (0.65-3.85)	0.316
GG	18 (6.55)	41 (7.72)	0.87 (0.48-1.57)	0.649	11 (9.32)	24 (8.54)	1.10 (0.50-2.39)	0.814	3 (8.33)	5 (6.94)	1.37 (0.29-6.50)	0.691
Additive			1.00 (0.79-1.26)	0.991			1.03 (0.74-1.43)	0.882			1.32 (0.70-2.50)	0.389
Dominant	127 (46.18)	240 (45.20)	1.04 (0.78-1.40)	0.775	59 (50.00)	139 (49.47)	1.01 (0.66-1.56)	0.961	16 (44.44)	26 (36.11)	1.54 (0.67-3.53)	0.314
Recessive	258 (93.45)	490 (92.28)	0.85 (0.48-1.50)	0.566	107 (90.68)	257 (91.46)	1.10 (0.52-2.33)	0.801	33 (91.67)	67 (93.06)	1.18 (0.26-5.38)	0.834
RAN rs14035 C>T												
CC	188 (68.36)	349 (65.73)	1.00		74 (62.71)	187 (66.55)	1.00		23 (63.89)	54 (75.00)	1.00	
CT	81 (29.45)	159 (29.94)	0.95 (0.69-1.31)	0.755	41 (34.75)	87 (30.96)	1.17 (0.74-1.86)	0.507	13 (36.11)	17 (23.61)	1.97 (0.80-4.87)	0.142
TT	6 (2.18)	23 (4.33)	0.48 (0.19-1.20)	0.117	3 (2.54)	7 (2.49)	1.17 (0.29-4.66)	0.828	0 (0.00)	1 (1.39)	/	/
Additive			0.85 (0.65-1.12)	0.248			1.14 (0.76-1.71)	0.516			1.74 (0.73-4.13)	0.213
Dominant	87 (31.64)	182 (34.27)	0.89 (0.65-1.22)	0.463	44 (37.29)	94 (33.45)	1.17 (0.75-1.84)	0.497	13 (36.11)	18 (25.00)	1.92 (0.78-4.73)	0.159
Recessive	269 (97.82)	508 (95.67)	0.49 (0.20-1.21)	0.123	115 (97.46)	274 (97.51)	1.11 (0.28-4.39)	0.884	36 (100.00)	71 (98.61)	/	/
RANBP2 rs2462788 C>T												
CC	258 (93.83)	489 (92.09)	1.00		112 (94.92)	256 (91.10)	1.00		32 (88.89)	65 (90.28)	1.00	
CT	17 (6.18)	42 (7.91)	0.76 (0.43-1.37)	0.364	6 (5.08)	25 (8.90)	0.56 (0.22-1.40)	0.214	4 (11.11)	7 (9.72)	1.22 (0.33-4.52)	0.770
TT	0 (0.00)	0 (0.00)	/	/	0 (0.00)	0 (0.00)	/	/	0 (0.00)	0 (0.00)	/	/
Additive			0.76 (0.43-1.37)	0.364			0.56 (0.22-1.40)	0.214			1.22 (0.33-4.52)	0.770
Dominant	17 (6.18)	42 (7.91)	0.76 (0.43-1.37)	0.364	6 (5.08)	25 (8.90)	0.56 (0.22-1.40)	0.214	4 (11.11)	7 (9.72)	1.22 (0.33-4.52)	0.770
Combined effect of protective genotypes for *RAN* [b]												
0	256 (93.09)	490 (92.28)	1.00		105 (88.98)	257 (91.46)	1.00		33 (91.67)	67 (93.06)	1.00	
1	12 (4.36)	17 (3.20)	1.38 (0.65-2.93)	0.406	11 (9.32)	17 (6.05)	1.56 (0.70-3.45)	0.275	3 (8.33)	4 (5.56)	1.40 (0.29-6.84)	0.676
2	3 (1.09)	7 (1.32)	0.79 (0.20-3.08)	0.729	2 (1.69)	0 (0.00)	/	/	0 (0.00)	0 (0.00)	/	/
3	4 (1.45)	17 (3.20)	0.46 (0.15-1.38)	0.165	0 (0.00)	7 (2.49)	/	/	0 (0.00)	1 (1.39)	/	/
0-2	271 (98.55)	514 (96.80)	1.00		118 (100.00)	274 (97.51)	1.00		36 (100.00)	71 (98.61)	1.00	
3	4 (1.45)	17 (3.20)	0.45 (0.15-1.34)	0.150	0 (0.00)	7 (2.49)	/	/	0 (0.00)	1 (1.39)	/	/

AOR, adjusted odds ratio; CI, confidence interval.

[a] Adjusted for age and gender.

[b] Protective genotypes were rs56109543 TT, rs7132224 GG and rs14035 TT from combined subjects.

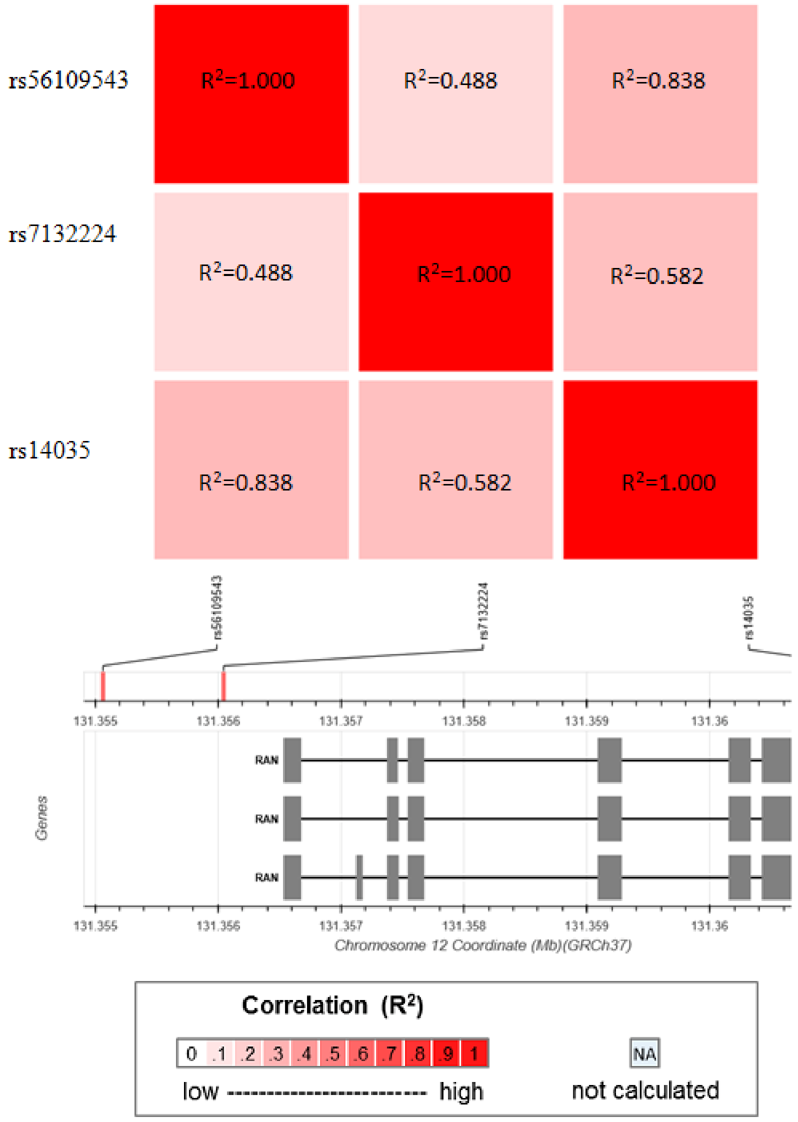

Supplementary Figure 1. Linkage disequilibrium analysis for the three selected polymorphisms in the RAN gene in Han Chinese population consisting of CHB (Han Chinese in Beijing, China) and CHS (Southern Han Chinese) subjects.
